# Effects on Fatty Acid Metabolism of a New Powdered Human Milk Fortifier Containing Medium-Chain Triacylglycerols and Docosahexaenoic Acid in Preterm Infants

**DOI:** 10.3390/nu10060690

**Published:** 2018-05-29

**Authors:** Claude Billeaud, Carole Boué-Vaysse, Leslie Couëdelo, Philippe Steenhout, Jonathan Jaeger, Cristina Cruz-Hernandez, Laurent Ameye, Jacques Rigo, Jean-Charles Picaud, Elie Saliba, Nicholas P. Hays, Frédéric Destaillats

**Affiliations:** 1 CIC Pédiatrique 1401 CHU, 33000 Bordeaux, France; claude.billeaud@chu-bordeaux.fr; 2 ITERG, Université de Bordeaux, 33076 Bordeaux, France; c.vaysse@iterg.com (C.B.-V.); l.couedelo@iterg.com (L.C.); 3 Nestlé Health Sciences, 1066 Epalinges, Switzerland; philippe.steenhout@nestle.com; 4 Nestlé Research Centre, 1000 Lausanne, Switzerland; jnthn.jaeger@gmail.com (J.J.); cristina.cruz-hernandez@rdls.nestle.com (C.C.-H.); 5 Nestlé Nutrition R&D, 1800 Vevey, Switzerland; laurent.ameye@nestle.com (L.A.); nicholaspaul.hays@nestle.com (N.P.H.); 6 Department of Neonatology, University of Liège, 4000 Liège, Belgium; j.rigo@ulg.ac.be; 7 Hôpital de la Croix Rousse, Hospices Civils, 69004 Lyon, France; jean-charles.picaud@chu-lyon.fr; 8 Hôpital Clocheville, CHU de Tours, 37004 Tours, France; elie.saliba@univ-tours.fr

**Keywords:** arachidonic acid, docosahexaenoic acid, fatty acid metabolism, medium-chain fatty acids, preterm infants

## Abstract

Preterm infants require fortification of human milk (HM) with essential fatty acids (FA) to ensure adequate post-natal development. As part of a larger randomized controlled study, we investigated FA metabolism in a subset of 47 clinically stable preterm infants (birth weight ≤1500 g or gestational age ≤32 weeks). Infants were randomized to receive HM supplemented with either a new HM fortifier (nHMF; *n* = 26) containing 12.5 g medium-chain FA (MCFA), 958 mg linoleic acid (LA), 417 mg α-linolenic acid (ALA), and 157 mg docosahexaenoic acid (DHA) per 100 g of powder (in compliance with the latest guidelines) or a fat-free HMF (cHMF; *n* = 21). Plasma phospholipid (PL) and triacylglycerol (TAG), and red blood cell phosphatidylcholine (RBC-PC) and phosphatidylethanolamine (RBC-PE) FA profiles were assessed before and after 21 days of feeding. In the nHMF group, significantly increased levels of *n*-9 monounsaturated fatty acids were observed, formed most likely by elongation and desaturation of dietary saturated fatty acids present in HM. ALA fortification increased ALA assimilation into plasma TAG. Similarly, DHA fortification enriched the DHA content in RBC-PE, which, in this compartment, was not associated with lower arachidonic acid levels as observed in plasma TAG and phospholipids. RBC-PE, a reliable indicator of FA metabolism and accretion, was the most sensitive compartment in this study.

## 1. Introduction

Omega-3 (*n*-3) and omega-6 (*n*-6) long-chain polyunsaturated fatty acids (LC-PUFAs) are of crucial importance in early life as they are essential for normal neurodevelopment and growth [1,2]. The most biologically active LC-PUFAs are docosahexaenoic acid (DHA; 22:6 *n*-3) and arachidonic acid (ARA; 20:4 *n*-6), which are synthesized from their precursors, α-linolenic acid (ALA; 18:3 *n*-3) and linoleic acid (LA; 18:2 *n*-6), respectively. Because ALA and LA cannot be synthesized de novo by mammalian cells, they are considered essential fatty acids and therefore must be supplied by the diet. During pregnancy, LC-PUFAs must be derived from maternal sources by placental transfer, and after birth they can only be obtained through the infant diet [3]. LC-PUFAs are essentially accumulated in the central nervous system and other tissues during the third trimester of pregnancy. Infants who are born prematurely are therefore deprived of this rapid in utero accretion and are considered at great risk of LC-PUFA deficiency [1,3,4]. LC-PUFA concentrations in the infant’s adipose tissue are insufficient to meet the requirements of the preterm infant and consequently LC-PUFAs required for organ growth are supplied by nutrition, intestinal absorption and conversion from precursor fatty acids [5]. While human milk is considered as the preferred source of nutrition for preterm infants compared to pre-term formula [6], it is also acknowledged that HM provides inadequate protein and micronutrients to support the rapid growth and bone mineralization of preterm infants. HM contains LA, ALA, DHA and ARA; however, the considerable variability of the DHA and ARA content means it may be inadequate for the requirements of preterm infants [5,7]. Consequently, fortification of human milk with essential fatty acids and LC-PUFAs is critical to achieve requirements and to satisfy the considerable demands of growth in the preterm infant [6,8]. Combining LC-PUFAs with human-milk fortifiers that increase the calorie, protein and mineral content of human milk has been proposed as a novel approach to supply LC-PUFAs to preterm infants [1]. There are various products used to enrich human milk, including multicomponent fortifiers. To date in Europe, the energy source of multicomponent fortifiers was exclusively carbohydrates. Recently, however, a novel powdered multicomponent fortifier was developed that provides both fats and carbohydrates as energy sources as well as increased levels of protein. The lipid component of this fortifier consists of medium-chain triacylglycerols (MCT) to provide an easily absorbed energy supply, and LC-PUFAs such as DHA, plus additional LA and ALA. We recently demonstrated in a randomized clinical study (http://ClinicalTrials.gov NCT01771588) that this new human milk fortifier (nHMF) is safe, well tolerated, and improves the weight gain of preterm infants, when compared to a control fat-free fortifier (cHMF) [9]. As part of this study, we conducted secondary experiments in a subset of infants to determine the baseline fatty acid composition of unfortified human milk and to compare the effects of nHMF and cHMF on fatty acid metabolism in preterm infants.

## 2. Materials and Methods

### 2.1. Study Design and Composition of the Control and New Human Milk Fortifiers

A randomized, double-blind, multicentre (11 European sites), controlled, parallel-group clinical trial was conducted in clinically stable preterm infants (gestational age ≤32 weeks or birth weight ≤1500 g) born to mothers who had elected to provide breast milk. Study design, participant characteristics, assessments and objectives have previously been presented in detail [9]. Each subject’s parent/legal representative gave their written informed consent for inclusion before participating in the study. The study was conducted in accordance with the Declaration of Helsinki, and the protocol was approved by the Ethics Committee at each study site (initial approval received from Kantonsapotheker Ethikkommission des Kantons Luzern (project identification code EK:1021) on 3 November 2010). Infants tolerating ≥100 mL/kg/day of human milk for >24 h were randomized to receive human milk fortified with cHMF or nHMF until discharge from the neonatal unit or medical decision (minimum 21 days). The composition of the two powdered HMF products is presented in Table 1. To achieve fortification, 5 g of cHMF or 4 g of nHMF were added to 100 mL of human milk. Fortifiers were initially given at half-strength, then increased according to hospital practice, with full-strength fortification occurring once infants were able to ingest 150–180 mL/kg/day (i.e., full enteral feeds; study Day 1). As described elsewhere [9], sample size was calculated to detect a non-inferior weight gain in infants fed with nHMF versus cHMF from D1 to D21 (non-inferiority margin—1 g/day, expected weight gain difference 2 g/day, standard deviation 4.73 g/day, type I error 5%, power 80%) [9]; 192 subjects (males and females combined) were needed. Group coding was used with two nonspeaking codes per group; fortifier packaging was coded accordingly but otherwise identical in appearance. Infants were enrolled and assigned to their intervention by the study investigators or trained delegates [9]. As part of this study, secondary experiments in a subset of infants were also conducted: in one single centre, fatty acid content was measured in human milk samples, while in three selected centres, lipid status was assessed from blood samples collected on Day 1 and Day 21 of full HM fortification.

### 2.2. Breast Milk and Infant Blood Collection

In order to characterize the fatty acid content of unfortified and fortified human milk, volunteer mothers of term infants provided breast-milk samples (*n* = 9). Samples were collected after full expression from one breast using a milk pump and while the baby was fed on the other breast. Every effort was made to collect representative samples of a complete feed including fore-, mid-, and hind-milk thus avoiding within-feed variations of lipid and other nutrient levels. Samples were stored at −80 °C and shipped on dry ice for analysis at the Nestlé Research Centre, Lausanne, Switzerland. A 25 mL aliquot was mixed with cHMF (4 g per 100 mL) or nHMF (5 g per 100 mL) or analysed as an unfortified sample. Blood samples (0.7 mL) were collected in EDTA-containing vacutainers from infants during Day 1 and again on Day 21 of full HM fortification. The blood was immediately centrifuged for 10 min at 1300× *g*, and plasma and red blood cells (RBC) were stored in microtubes at −80 °C until analysis.

### 2.3. Fatty Acid Analysis

#### 2.3.1. Breast Milk Fatty Acid Methyl Ester Preparation and Analysis

Fatty acids were analysed as fatty acid methyl esters (FAMEs) according to the technique recommended by Cruz-Hernandez et al. [10]. The methylation procedure was as follows: 250 µL of human milk, then 300 µL of internal standard FAME 11:0 and 300 µL of internal standard TAG 13:0, 2 mL of methanol, 2 mL of methanol/HCl (3 M) and 1 mL of *n*-hexane were added to a 15 mL test tube with a Teflon-lined screw cap. Test tubes were tightly capped, shaken vigorously, then heated for 60 min at 100 °C, during which time they were occasionally shaken. Great care was taken to ensure caps were tightly sealed with the cap liner to avoid leaks during heating. The test tubes were cooled to room temperature, then 2 mL of water were added. The tubes were then shaken vigorously before centrifugation at 1200*× g* for 5 min, after which the upper phase (*n*-hexane) was transferred into gas chromatography (GC) vials. GC analyses were conducted using a 7890 A gas chromatograph with a 7693 autosampler equipped with a preparative station module (Agilent Technologies, Palo Alto, CA, USA) fitted with a fused-silica CP-Sil 88 capillary column (100% cyanopropylpolysiloxane; 100 m, 0.25 mm ID, 0.25 mm film thickness; Agilent Technologies, Palo Alto, CA, USA) and a split injector (1:25 ratio) heated to 250 °C and a flame ionization detector operated at 300 °C. The oven temperature was programmed at 60 °C isothermal for 5 min, increased by 15 °C/min to 165 °C, isothermal for 1 min at this temperature, then increased to 195 °C by 2 °C/min and held isothermal for 14 min, and then increased to 215 °C by 5 °C/min and held isothermal for 8 min at 215 °C. Hydrogen was used as carrier gas in constant flow mode at 1.5 mL/min.

#### 2.3.2. Plasma Lipid Class Separation

Lipids were extracted from plasma according to the technique recommended by Folch et al. [11]. Lipid classes were separated by thin-layer chromatography (TLC) and sample migration was performed with hexane/diethyl ether/acetic acid (80/20/1; *v*/*v*/*v*). After drying, the lipid classes were visualized by spraying the TLC plate with 1,2-dichlorofluorescein and detecting under UV-light. The lipid fractions (PL and TAG) were identified by comparison with standards and collected in glass tubes. Standard TAG 17:0 and PE 17:0 were added to the TAG and PL extracts, respectively.

#### 2.3.3. RBC Phospholipid Class Separation

Lipids were extracted from the RBC according to the method recommended by Peuchant [12]. PC and PE were separated from the RBC lipid extract by TLC and sample migration was performed with chloroform/methanol/acetic acid/water (50/37.5/3.5/2; *v*/*v*/*v*). PC and PE were visualized by spraying the TLC with 1,2-dichlorofluorescein and detecting under UV-light. The lipid fractions from PC and PE were identified by comparison with standards then collected in glass tubes. Standard PE and PC 17:0 were added to the PE and PC extracts, respectively.

#### 2.3.4. Plasma and RBC FAME Preparation and Analysis

Fatty acids in plasma TAG and PL were transesterified according to the method described by Morrison and Smith [13]. FAMEs were analysed by GC on a BPX 70 capillary column (60 m long, 0.25 µm film, 0.25 mm ID, SGE, Milton Keynes, UK). Hydrogen was used as carrier gas with a constant 120 kPa pressure and flow of 20 mL/min. The GC system consisted of a Focus GS (Thermofinnigan, Courtaboeuf, France) equipped with a split injector (1:80 ratio) heated to 250 °C and a flame ionization detector operated at 250 °C. The column temperature was increased from 150 °C to 200 °C (1.3 °C/min), maintained at 200 °C for 20 min, increased from 200 °C to 235 °C (10 °C/min), and held at 235 °C for 20 min. ChromQuest software (Thermofinnigan, Courtaboeuf, France) was used for data acquisition and handling. A pure FAME mixture (Sigma, St Louis, MO, USA) of known composition was used as the standard for column calibration. The variation in peak area between injections was below 2%.

### 2.4. Statistical Analysis

Levels of fatty acids were measured at Day 1 and Day 21 of full HM fortification, in plasma PL, plasma TAG, RBC-PC and RBC-PE. Summary statistics for each fatty acid and for each compartment were calculated at each visit. Data distribution was close to log-normal; geometric mean and geometric standard deviation are provided in the present paper instead of arithmetic mean and standard deviation. Relative fatty acid concentrations were analysed at Day 21 (log-transformation) using a mixed-effect ANCOVA model adjusted for postmenstrual age at Day 1, weight at Day 1, fatty acid concentration at Day 1, sex, centre and treatment group (with centre considered as a random effect). Estimations of the treatment effect nHMF/cHMF (log of the ratio of the geometric estimates) and the two-sided *p*-value are given in for each fatty acid analysed in the different lipid compartments.

## 3. Results

### 3.1. Study Population

As reported elsewhere [9], 153 premature infants were enrolled in this clinical study and randomized to receive nHMF (*n* = 77) or cHMF (*n* = 76). Infant demographic and baseline anthropometric characteristics and the number of twins were similar in both groups [9]. Fatty acid profiles in plasma PL and TAG, RBC-PC and RBC-PE were analysed in a subset of 47 infants (*n* = 21 and *n* = 26 fed with cHMF and nHMF, respectively).

### 3.2. Fatty Acids in Human Milk

The quantitative fatty acid profile of nine samples of human milk was determined. Table 2 shows the fatty acid composition (mg per 100 mL) of unfortified human milk and of human milk fortified with cHMF or nHMF. Of the medium-chain FA measured, 8:0 and 10:0 were increased in human milk fortified with nHMF when compared to cHMF due to the use of medium-chain triglyceride oil in the nHMF. LA, ALA, EPA and DHA levels were also greater (nearly twice) in the nHMF than in the cHMF. According to the estimated levels of essential and LC-PUFAs provided by human milk fortified with cHMF or nHMF in preterm infants (Table 3), human milk fortified with nHMF provided the recommended intakes of essential fatty acids and LC-PUFAs for premature infants [6,14,15]. It is important to note that the limited number of samples (*n* = 9) does not allow estimation of the variable composition of the fortified HM, resulting from natural fluctuation of macronutrient content and composition (i.e., lipids), or impact on processing steps such as HM collection, handling and pasteurization.

### 3.3. Fatty Acid Profile in Plasma and RBC Lipids of Preterm Infants Fed cHMF or nHMF

The fatty acid profile (expressed as g per 100 g of fatty acids) of plasma PL, plasma TAG, RBC-PC and RBC-PE fractions in the two study groups (nHMF and cHMF), before and after 21 days of feeding, are reported in Table 4, Table 5, Table 6 and Table 7 respectively.

Table 4 shows that after 21 days of feeding, saturated fatty acid levels in the plasma PL fraction were comparable in both study groups, with the exception of significantly higher 16:0 and significantly lower 18:0 dimethyl acetal (DMA) in the nHMF compared to the cHMF group. 18:1 *n*-7 was the only monounsaturated fatty acid that was significantly increased in the nHMF group, compared to the cHMF group. Of the PUFAs, 20:3 *n*-6 (dihomo-γ-linolenic acid; DGLA), 20:4 *n*-6 (ARA), 22:4 *n*-6, 22:5 *n*-3 (*n*-3 docosapentaenoic acid (*n*-3 DPA)) levels were significantly lower, whereas 20:5 *n*-3 (eicosapentaenoic acid; EPA) levels were significantly higher in the nHMF compared to the cHMF group. LA, ALA and DHA levels were not significantly different between the two study groups.

In the plasma TAG compartment (Table 5), saturated fatty acid levels did not differ between the two groups after 21 days of dietary intervention. Compared to the cHMF group, the nHMF group showed significant variations in levels of two monounsaturated fatty acids, *trans-*18.1 and 20:1 *n*-9. With regard to PUFAs, the nHMF group was characterized by a significant increase in ALA and a significant decrease in ARA, 22:4 *n*-6 and *n*-3 DPA, when compared to the cHMF group. LA and DHA levels did not differ significantly between the two groups.

In the RBC-PC compartment, on Day 21 of the study, the nHMF group showed a significant increase in 18:1 *n*-7, 16:1 *n*-9 and 18:1 *n*-9, and a significant decrease in 18:0 levels, compared to the cHMF group (Table 6). No differences were found between groups for the PUFAs.

In the RBC-PE compartment (Table 7), 21 days of dietary intervention with nHMF were associated with a significant decrease in levels of two saturated fatty acids (15:0, 16:0) and a significant increase in levels of two monounsaturated fatty acids (18:1 *n*-7, 20:1 *n*-9), when compared to the cHMF intervention. A significant increase in levels of several PUFAs, DGLA, EPA, *n*-3 DPA, and DHA was also observed in the nHMF group. No differences in ARA levels were observed between the two groups.

## 4. Discussion

**Figure 1:**
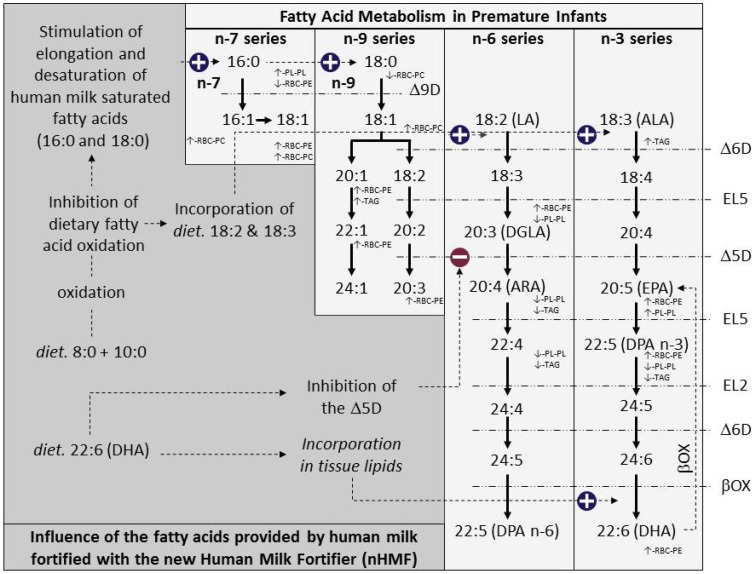
Schematic representation of the influence of dietary fatty acids provided by the nHMF. Differences (shown by the ↑ and ↓ symbols) between relative concentrations of FA provided by the nHMF or the cHMF in the compartments assessed are statistically significant (*p* <0.05). Metabolic pathways responsible for the biosynthesis of FA from the *n*-3, *n*-6, *n*-7, and *n*-9 series are displayed together with the enzymes involved (Δ9D, Δ9-desaturase; Δ6D, Δ6-desaturase, EL5, elongase 5; Δ5D, Δ5-desaturase; EL2, elongase 2; βOX, β-oxidation).

The lipid composition of the nHMF is intended to provide significant quantities of dietary MCFA 8:0 and 10:0, ALA and DHA, as a complement to HM innate lipid content (Table 2). In the present study, a subset of HM samples was fortified with nHMF to validate both its addition rate and composition in accordance to the current recommendations (Table 3). It is important to note that large inter-individual variation of ARA and DHA content in HM has been observed mainly due to the influence of maternal diet [7]. In the present study, comparison of the fatty acid profile of erythrocyte and plasma lipids in infants fed HM fortified with nHMF or cHMF provides valuable insight into the fatty acid metabolism of preterm infants. Based on the full dataset, Figure 1 gives a comprehensive representation of the changes in fatty acid levels when preterm infants are fed HM fortified with the nHMF compared to preterm infants fed HM fortified with cHMF. Figure 1 also provides a putative explanation of the factors driving these changes. Almost all the fatty acids of the *n*-7, *n*-9, *n*-6 and *n*-3 series were impacted by the nHMF, thus confirming a highly active fatty acid metabolism in the preterm infants of our study (Figure 1). In the present study, an accumulation of metabolites formed by Δ9-desaturation and elongation products of saturated fatty acids, up to 22:1 *n*-9 in RBC-PE, is observed (Figure 1). Increased levels of elongation and desaturation metabolites formed from saturated fatty acids (e.g., palmitic and stearic acids) have been previously observed in rats fed with MCT and labelled palmitic and stearic acid [16]. In this animal experiment, Leveille et al. demonstrated that feeding 8:0 and 10:0 acids stimulates the elongation of exogenous palmitic acid as well as formation of oleic acid from stearic acid by Δ9-desaturation [16]. It can therefore be hypothesized that feeding MCFA provided by dietary MCT stimulates the metabolism of saturated fatty acids provided by human milk as represented schematically in Figure 1. Monounsaturated fatty acids are important components of polar lipids in the central nervous system and in particular in the myelin sheath [17,18]. Myelination begins during the last trimester of gestation, and nutrition in preterm infants plays a critical role in this process [19]. One can therefore hypothesize that supplying dietary lipids to stimulate the formation of monounsaturated fatty acids, and in particular *n*-9 long-chain monounsaturated fatty acids, during the neonatal period is an appealing nutritional strategy to support the myelination process in the preterm infant.

The use of essential fatty acids, and ALA in particular, in the composition of the nHMF led to an increased incorporation of ALA in plasma TAG of pre-term infants fed nHMF (Table 5 and Figure 1). Enhanced ALA intake (Table 2) led to greater accumulation of ALA metabolites (including EPA and *n*-3 DPA) formed by elongation and desaturation, and notably in RBC-PE (Table 7 and Figure 1). Dietary DHA provided by the nHMF led to beneficial increases in DHA levels in RBC-PE, which has been used as a proxy for neural tissue lipids [20]. LA levels were almost identical in nHMF and cHMF when mixed with HM (Table 2). Levels of LA and its fatty acid metabolites were therefore not enriched in the blood lipids of pre-term infants, with the one exception of higher levels of DGLA in the RBC-PE of infants fed nHMF (Figure 1). Slightly lower levels of LA metabolites, and DGLA, ARA and *n*-3 DPA in particular, were observed in plasma lipids, suggesting potential inhibition of LA metabolism by the pre-formed DHA provided to pre-term infants fed nHMF. However, such modulation of the *n*-6 fatty acids by dietary DHA was not observed in RBC-PE (Table 7). One may therefore hypothesize that the lower levels of *n*-6 fatty acids observed in plasma lipids do not impact the fatty acid composition of tissue lipids.

One important limitation of the present study is the limited duration of the feeding period (21 days). Therefore, the observed impact of nHMF on the composition of circulatory lipids and in particular on the RBC membrane lipids should be critically considered. It is known indeed, that RBC have a half-life longer than the observation period. Pre-term infants are also developing rapidly, and therefore it is difficult to predict the impact of the nHMF on circulatory lipids over a longer time of exposure.

## 5. Conclusions

The present study shows that fortifying human milk with a new fortifier containing MCT, as a source of MCFA, ALA and DHA led to an increased incorporation of *n*-9 long-chain monounsaturated fatty acids in RBC-PE, which is a reliable indicator of fatty acid metabolism in premature infants.

## Figures and Tables

**Table 1:** Nutritional composition of the control (cHMF) and new human milk fortifier (nHMF).

Nutrients (per 100 g of Powder) *	cHMF	nHMF
Protein (g)	20.00	35.50
Carbohydrates (g)	66.00	32.40
Lipid content (g) **	0.38	18.10
Saturated fatty acids (g)	-	12.20
Medium chain fatty acids (MCFA, g)	-	12.50
Monounsaturated fatty acids (g)	-	2.93
Linoleic acid (LA, mg)	-	958.00
α-Linolenic acid (ALA, mg)	-	417.00
Arachidonic acid (ARA, mg)	-	13.80
Docosahexaenoic acid (DHA, mg)	-	157.00

* Vitamins and minerals are not listed. ** Total fatty acid recovery of 0.92 g per 100 g of lipids (glycerol and unsaponifiable lipids not measured).

**Table 2:** Fatty acid content (mg/100 mL) of unfortified human milk * and human milk fortified with a control (cHMF) or a new human milk fortifier (nHMF), and level (%) of fortification achieved with nHMF relative to cHMF.

Fatty Acid	Human Milk (HM, *n* = 9)		HM Fortified with cHMF (*n* = 9)		HM Fortified with nHMF (*n* = 9)		Fortification ** (nHMF vs. cHMF)
	Mean	SD	Mean	SD	Mean	SD	%
6:0	2.1	0.9	2.1	1.2	2.2	1.4	-
8:0	8.3	2.5	10.5	3.4	243.8	17.5	2222
10:0	55.3	18.5	56.4	22.5	245.2	24.9	335
12:0	190.1	62.3	191.6	81.5	190.2	80.1	-
14:0	233.3	64.2	236.9	95.9	242.2	100.2	-
16:0	768.0	227.8	771.3	274.1	737.0	272.3	-
16:1 *n*-7	71.0	30.2	68.8	29.9	93.5	101.1	36
18:0	212.7	58.9	213.7	69.0	204.5	79.5	-
*trans*-18:1	25.0	11.2	24.7	13.0	32.3	33.8	31
18:1 *n*-9	1096.9	275.4	1079.7	277.9	1149.6	272.4	-
18:1 *n*-7	65.6	15.3	65.5	22.7	71.6	20.8	-
18:2 *n*-6 (LA)	317.6	105.7	311.1	92.3	336.2	92.5	-
18:3 *n*-3 (ALA)	29.5	10.7	28.5	8.8	43.8	10.2	54
18:3 *n*-6 (GLA)	2.6	1.4	2.7	1.6	2.6	1.3	-
20:0	6.0	2.2	6.2	2.4	6.8	2.2	10
20:1 *n*-9	15.7	4.7	15.7	5.3	17.4	5.6	11
20:2 *n*-6	9.8	3.8	10.1	4.1	10.0	4.0	-
20:3 *n*-6 (DGLA)	13.3	6.3	13.5	8.4	13.0	6.9	-
22:1 *n*-9	2.9	1.2	3.1	1.4	3.0	1.5	-
20:4 *n*-6 (ARA)	15.9	7.3	15.7	9.1	15.8	8.0	-
20:5 *n*-3 (EPA)	2.3	1.1	2.1	0.9	3.4	1.2	62
24:0	3.2	1.3	3.4	1.7	3.6	1.6	-
24:1 *n*-9	3.5	1.9	3.6	2.4	3.9	2.5	-
22:6 *n*-3 (DHA)	12.7	5.8	12.5	6.3	18.1	6.7	45

* Data based on analysis of 9 donor milk samples. ** Values reported ≥ 10%. ALA, α-linolenic acid; ARA, arachidonic acid; DGLA, dihomo-γ-linolenic acid; DHA, docosahexaenoic acid; EPA, eicosapentaenoic acid; GLA, γ-linolenic acid; LA, linoleic acid; SD, standard deviation.

**Table 3:** Estimated levels of essential and long-chain polyunsaturated fatty acids provided by human milk (HM) fortified with a control (cHMF) or a new human milk fortifier (nHMF) in preterm infant study participants (mg·kg^−1^·day^−1^). Comparison with recent expert panel recommendations [6,14,15].

Fatty Acid	Unit	HM * Fortified with		Recent Recommendations		
		cHMF	nHMF	ESPGHAN 2010 [15]	Lapillonne et al. 2014 [6]	Koletzko et al. 2014 [14]
18:2 *n*-6 (LA)	mg·kg^−1^·day^−1^	475	513	350–1400	350–1400	385–1540
18:3 *n*-3 (ALA)	mg·kg^−1^·day^−1^	44	67	>55	>55	>55
LA:ALA	-	11:1	8:1	5–15:1	5–15:1	-
20:4 *n*-6 (ARA)	mg·kg^−1^·day^−1^	24	24	18–42	18–45	18–45
22:6 *n*-3 (DHA)	mg·kg^−1^·day^−1^	19	28	12–30	12–60	18–60
ARA:DHA	-	1.3:1	0.8:1	1–2:1	-	-
20:5 *n*-3 (EPA)	mg·kg^−1^·day^−1^	3 **	5 ^†^	<30% DHA	<20	<20

* Estimation based on 9 donor human milk samples collected in the present study. ** 16% of DHA supply. ^†^ 18% of DHA supply. ALA, α-linolenic acid; ARA, arachidonic acid; DHA, docosahexaenoic acid; EPA, eicosapentaenoic acid; LA, linoleic acid.

**Table 4:** Fatty acid profile (g/100 g of fatty acids) of plasma total phospholipids in preterm infants receiving human milk fortified with a control (cHMF) or with a new human milk fortifier (nHMF), before and after 21 days of treatment. Estimations of the treatment effect nHMF/cHMF (Difference) and the two-sided *p*-value are given for each fatty acid analysed in the different lipid compartments.

Fatty Acid	cHMF (*n* = 21)				nHMF (*n* = 26)				Difference	*p* Value
	Baseline		after 21 Days		Baseline		after 21 Days			
	Mean	SD	Mean	SD	Mean	SD	Mean	SD		
14:0	0.10	0.04	0.17	0.08	0.14	0.08	0.18	0.09	−0.056	0.719
15:0	0.22	0.21	0.32	0.26	0.31	0.25	0.31	0.32	−0.611	0.024
16:0	15.30	2.09	16.47	3.00	16.37	2.38	15.71	2.98	−0.123	0.040
16:0 DMA	5.24	0.59	5.42	0.91	5.38	0.71	5.61	0.78	0.061	0.163
16:1 *n*-7	0.34	0.08	0.39	0.22	0.36	0.16	0.41	0.22	−0.039	0.699
16:1 *n*-9	0.31	0.09	0.36	0.11	0.34	0.12	0.38	0.17	−0.061	0.588
18:0	6.69	1.38	6.84	1.54	6.56	1.07	6.64	1.19	−0.019	0.735
18:0 DMA	9.30	1.31	8.62	1.29	9.28	1.20	8.45	1.44	0.007	0.871
18:1 DMA	3.77	0.63	3.55	0.56	3.37	0.63	3.54	0.63	0.094	0.051
18:1 *n*-7	1.25	0.17	1.29	0.26	1.15	0.23	1.40	0.35	0.114	0.013
18:1 *n*-9	15.49	1.67	14.31	1.42	14.65	1.54	14.67	1.39	0.029	0.243
*trans*-18:1	0.22	0.11	0.29	0.13	0.22	0.09	0.26	0.09	−0.074	0.585
18:2 *n*-6 (LA)	2.66	0.46	2.97	0.57	2.85	0.72	3.30	0.69	0.013	0.779
18:3 *n*-3 (ALA)	0.20	0.13	0.25	0.15	0.25	0.15	0.29	0.20	−0.139	0.414
18:3 *n*-6 (GLA)	0.09	0.03	0.09	0.03	0.09	0.03	0.11	0.04	0.118	0.263
20:0	0.09	0.04	0.09	0.05	0.09	0.05	0.10	0.05	0.059	0.723
20:1 *n*-9	0.06	0.03	0.08	0.05	0.06	0.04	0.09	0.07	−0.011	0.958
20:2 *n*-6	0.50	0.11	0.56	0.14	0.43	0.14	0.60	0.17	0.174	0.003
20:3 *n*-6 (DGLA)	0.17	0.05	0.21	0.05	0.17	0.06	0.20	0.06	0.012	0.879
20:3 *n*-9	1.76	0.27	1.80	0.41	1.56	0.24	1.84	0.25	0.099	0.031
20:4 *n*-6 (ARA)	1.33	0.35	1.58	0.89	1.22	0.49	1.95	1.10	0.247	0.011
20:5 *n*-3 (EPA)	20.78	1.75	20.54	2.25	21.13	1.42	19.65	2.32	−0.024	0.374
22:0	0.79	0.44	0.68	0.22	0.71	0.31	0.95	0.18	0.301	<0.001
22:1 *n*-9	0.03	0.02	0.04	0.03	0.03	0.02	0.05	0.03	−0.25	0.193
22:4 *n*-6	0.07	0.03	0.07	0.03	0.05	0.03	0.07	0.03	0.352	0.008
22:5 *n*-3 (*n*-3 DPA)	4.75	0.71	4.78	0.65	4.77	0.66	4.48	0.61	−0.036	0.349
22:5 *n*-6 (*n*-6 DPA)	1.46	0.67	1.48	0.42	1.31	0.54	1.65	0.35	0.117	0.019
22:6 *n*-3 (DHA)	0.97	0.34	1.00	0.33	0.90	0.22	0.95	0.19	0.047	0.327
24:0	5.64	1.00	5.14	0.91	5.76	0.81	5.61	1.17	0.092	0.016
24:1 *n*-9	0.05	0.02	0.04	0.02	0.04	0.02	0.04	0.03	−0.201	0.351

Data are presented as geometric mean and geometric standard deviation (SD). ALA, α-linolenic acid; ARA, arachidonic acid; DGLA, dihomo-γ-linolenic acid; DHA, docosahexaenoic acid; DMA, dimethyl acetal; DPA, docosapentaenoic acid; EPA, eicosapentaenoic acid; GLA, γ-linolenic acid; LA, linoleic acid.

**Table 5:** Fatty acid profile (g/100 g of fatty acids) of plasma triacylglycerols in preterm infants receiving human milk fortified with a control (cHMF) or with a new human milk fortifier (nHMF), before and after 21 days of treatment. Estimations of the treatment effect nHMF/cHMF (Difference) and the two-sided *p*-value are given for each fatty acid analysed in the different lipid compartments.

Fatty Acid	cHMF (*n* = 21)				nHMF (*n* = 26)				Difference	*p* Value
	Baseline		after 21 Days		Baseline		after 21 Days			
	Mean	SD	Mean	SD	Mean	SD	Mean	SD		
14:0	2.23	1.09	2.22	1.38	2.77	1.40	2.56	1.36	0.243	0.262
*iso*-16:0	0.05	0.02	0.05	0.02	0.06	0.02	0.06	0.02	0.203	0.259
15:0	0.26	0.12	0.27	0.06	0.27	0.07	0.28	0.08	−0.013	0.868
16:0	25.83	1.90	26.96	3.65	28.38	4.62	29.89	4.02	0.052	0.065
16:1 *n*-7	6.50	2.37	4.89	2.77	5.99	2.26	5.11	2.40	0.100	0.331
16:1 *n*-9	0.96	0.26	0.87	0.24	0.95	0.23	0.93	0.26	0.087	0.226
17:1	0.25	0.04	0.23	0.04	0.24	0.04	0.25	0.06	0.078	0.176
18:0	3.73	0.79	4.68	0.84	4.22	1.17	4.77	0.74	−0.032	0.529
18:1 *n*-7	4.71	1.39	4.12	1.56	4.31	1.35	4.32	1.66	0.065	0.371
18:1 *n*-9	43.18	2.50	40.22	3.91	39.68	4.39	38.41	3.33	−0.018	0.517
*trans*-18:1	0.35	0.13	0.53	0.16	0.47	0.21	0.42	0.15	−0.289	0.012
18:2 *n*-6 (LA)	6.27	2.48	8.58	3.73	7.09	3.52	7.15	3.59	−0.135	0.238
18:3 *n*-3 (ALA)	0.45	0.25	0.56	0.24	0.73	0.36	0.75	0.32	0.346	0.003
18:3 *n*-6 (GLA)	0.23	0.06	0.22	0.08	0.21	0.07	0.19	0.06	−0.095	0.249
20:0	0.11	0.03	0.13	0.05	0.11	0.04	0.13	0.04	−0.008	0.941
20:1 *n*-7	0.14	0.05	0.16	0.07	0.13	0.05	0.13	0.05	−0.105	0.363
20:1 *n*-9	0.47	0.07	0.51	0.09	0.49	0.10	0.54	0.07	0.069	0.042
20:2 *n*-6	0.18	0.08	0.22	0.09	0.20	0.07	0.19	0.10	−0.147	0.232
20:3 *n*-6 (DGLA)	0.27	0.08	0.32	0.13	0.25	0.11	0.25	0.15	−0.221	0.075
20:3 *n*-9	0.72	0.34	0.54	0.27	0.49	0.20	0.50	0.19	0.091	0.435
20:4 *n*-6 (ARA)	0.83	0.25	0.99	0.50	0.73	0.30	0.71	0.48	−0.299	0.029
20:5 *n*-3 (EPA)	0.14	0.09	0.16	0.13	0.17	0.12	0.16	0.08	0.176	0.158
22:4 *n*-6	0.13	0.04	0.16	0.07	0.13	0.04	0.11	0.06	−0.298	0.021
22:5 *n*-3 (DPA)	0.17	0.09	0.20	0.13	0.16	0.09	0.15	0.09	−0.237	0.042
22:5 *n*-6 (DPA)	0.23	0.09	0.30	0.17	0.18	0.08	0.24	0.13	−0.070	0.662
22:6 *n*-3 (DHA)	0.86	0.68	1.08	0.87	0.78	0.52	0.98	0.79	0.092	0.472

Data are presented as geometric mean and geometric standard deviation (SD). ALA, α-linolenic acid; ARA, arachidonic acid; DGLA, dihomo-γ-linolenic acid; DHA, docosahexaenoic acid; DMA, dimethyl acetal; DPA, docosapentaenoic acid; EPA, eicosapentaenoic acid; GLA, γ-linolenic acid; LA, linoleic acid.

**Table 6:** Fatty acid profile (g/100 g of fatty acids) of red blood cell phosphatidylcholine (RBC PC) in preterm infants receiving human milk fortified with a control (cHMF) or with a new human milk fortifier (nHMF), before and after 21 days of treatment. Estimations of the treatment effect nHMF/cHMF (Difference) and the two-sided *p*-value are given for each fatty acid analysed in the different lipid compartments.

Fatty Acid	cHMF (*n* = 21)				nHMF (*n* = 26)				Difference	*p* Value
	Baseline		after 21 Days		Baseline		after 21 Days			
	Mean	SD	Mean	SD	Mean	SD	Mean	SD		
14:0	0.75	0.27	0.90	0.37	0.67	0.27	0.82	0.30	0.203	0.164
15:0	0.27	0.09	0.31	0.09	0.27	0.09	0.33	0.08	0.131	0.185
16:0	43.91	3.45	44.01	3.36	43.87	3.91	44.23	3.34	0.035	0.165
16:0 DMA	0.35	0.11	0.32	0.09	0.37	0.11	0.32	0.10	0.044	0.655
16:1 *n*-7	2.02	0.87	1.54	1.38	2.02	1.05	1.35	0.83	0.093	0.515
16:1 *n*-9	0.57	0.13	0.47	0.23	0.49	0.15	0.49	0.19	0.215	0.034
18:0	7.28	1.66	8.95	2.65	7.81	1.51	8.21	1.38	−0.179	0.010
18:0 DMA	0.10	0.04	0.08	0.03	0.09	0.03	0.07	0.03	−0.065	0.636
18:1 DMA	0.13	0.04	0.13	0.03	0.15	0.07	0.14	0.05	−0.013	0.891
18:1 *n*-7	3.60	0.49	3.34	0.59	3.71	0.65	3.56	0.63	0.101	0.038
18:1 *n*-9	22.74	2.11	20.89	2.49	22.19	2.63	21.86	1.94	0.068	0.038
*trans*-18:1	0.14	0.08	0.27	0.12	0.17	0.07	0.26	0.11	−0.122	0.383
18:2 *n*-6 (LA)	10.48	2.21	11.47	2.26	11.22	2.14	11.53	2.09	−0.011	0.868
18:3 *n*-3 (ALA)	0.17	0.09	0.24	0.17	0.22	0.19	0.21	0.11	−0.322	0.113
18:3 *n*-6 (GLA)	0.16	0.06	0.11	0.03	0.15	0.07	0.11	0.03	−0.015	0.878
20:0	0.10	0.05	0.11	0.05	0.10	0.04	0.11	0.04	0.061	0.673
20:1 *n*-9	0.28	0.09	0.31	0.10	0.27	0.12	0.32	0.05	0.128	0.294
20:2 *n*-6	0.23	0.04	0.32	0.08	0.26	0.06	0.33	0.07	−0.040	0.629
20:3 *n*-6 (DGLA)	1.61	0.49	1.66	0.55	1.52	0.50	1.52	0.51	−0.145	0.232
20:3 *n*-9	0.48	0.35	0.39	0.24	0.37	0.22	0.41	0.25	−0.124	0.536
20:4 *n*-6 (ARA)	3.13	1.99	2.62	1.58	2.52	1.50	2.22	1.50	−0.286	0.237
20:5 *n*-3 (EPA)	0.22	0.17	0.15	0.13	0.22	0.19	0.17	0.14	0.011	0.962
22:4 *n*-6	0.15	0.11	0.14	0.08	0.13	0.07	0.12	0.06	−0.091	0.688
22:6 *n*-3 (DHA)	0.37	0.31	0.43	0.43	0.36	0.32	0.39	0.32	−0.252	0.395

Data are presented as geometric mean and geometric standard deviation (SD). ALA, α-linolenic acid; ARA, arachidonic acid; DGLA, dihomo-γ-linolenic acid; DHA, docosahexaenoic acid; DMA, dimethyl acetal; DPA, docosapentaenoic acid; EPA, eicosapentaenoic acid; GLA, γ-linolenic acid; LA, linoleic acid.

**Table 7:** Fatty acid profile (g/100 g of fatty acids) of red blood cell phosphatidylethanolamine (RBC PE) in preterm infants receiving human milk fortified with a control (cHMF) or with a new human milk fortifier (nHMF), before and after 21 days of treatment. Estimations of the treatment effect nHMF/cHMF (Difference) and the two-sided *p*-value are given for each fatty acid analysed in the different lipid compartments.

Fatty Acid	cHMF (*n* = 21)				nHMF (*n* = 26)				Difference	*p* Value
	Baseline		after 21 Days		Baseline		after 21 Days			
	Mean	SD	Mean	SD	Mean	SD	Mean	SD		
14:0	0.10	0.04	0.17	0.08	0.14	0.08	0.18	0.09	−0.056	0.719
15:0	0.22	0.21	0.32	0.26	0.31	0.25	0.31	0.32	−0.611	0.024
16:0	15.30	2.09	16.47	3.00	16.37	2.38	15.71	2.98	−0.123	0.04
16:0 DMA	5.24	0.59	5.42	0.91	5.38	0.71	5.61	0.78	0.061	0.163
16:1 *n*-7	0.34	0.08	0.39	0.22	0.36	0.16	0.41	0.22	−0.039	0.699
16:1 *n*-9	0.31	0.09	0.36	0.11	0.34	0.12	0.38	0.17	−0.061	0.588
18:0	6.69	1.38	6.84	1.54	6.56	1.07	6.64	1.19	−0.019	0.735
18:0 DMA	9.30	1.31	8.62	1.29	9.28	1.20	8.45	1.44	0.007	0.871
18:1 DMA	3.77	0.63	3.55	0.56	3.37	0.63	3.54	0.63	0.094	0.051
18:1 *n*-7	1.25	0.17	1.29	0.26	1.15	0.23	1.40	0.35	0.114	0.013
18:1 *n*-9	15.49	1.67	14.31	1.42	14.65	1.54	14.67	1.39	0.029	0.243
*trans*-18:1	0.22	0.11	0.29	0.13	0.22	0.09	0.26	0.09	−0.074	0.585
18:2 *n*-6 (LA)	2.66	0.46	2.97	0.57	2.85	0.72	3.30	0.69	0.013	0.779
18:3 *n*-3 (ALA)	0.20	0.13	0.25	0.15	0.25	0.15	0.29	0.20	−0.139	0.414
18:3 *n*-6 (GLA)	0.09	0.03	0.09	0.03	0.09	0.03	0.11	0.04	0.118	0.263
20:0	0.09	0.04	0.09	0.05	0.09	0.05	0.10	0.05	0.059	0.723
20:1 *n*-7	0.06	0.03	0.08	0.05	0.06	0.04	0.09	0.07	−0.011	0.958
20:1 *n*-9	0.50	0.11	0.56	0.14	0.43	0.14	0.60	0.17	0.174	0.003
20:2 *n*-6	0.17	0.05	0.21	0.05	0.17	0.06	0.20	0.06	0.012	0.879
20:3 *n*-6 (DGLA)	1.76	0.27	1.80	0.41	1.56	0.24	1.84	0.25	0.099	0.031
20:3 *n*-9	1.33	0.35	1.58	0.89	1.22	0.49	1.95	1.10	0.247	0.011
20:4 *n*-6 (ARA)	20.78	1.75	20.54	2.25	21.13	1.42	19.65	2.32	−0.024	0.374
20:5 *n*-3 (EPA)	0.79	0.44	0.68	0.22	0.71	0.31	0.95	0.18	0.301	<0.001
22:0	0.03	0.02	0.04	0.03	0.03	0.02	0.05	0.03	−0.25	0.193
22:1 *n*-9	0.07	0.03	0.07	0.03	0.05	0.03	0.07	0.03	0.352	0.008
22:4 *n*-6	4.75	0.71	4.78	0.65	4.77	0.66	4.48	0.61	−0.036	0.349
22:5 *n*-3 (DPA)	1.46	0.67	1.48	0.42	1.31	0.54	1.65	0.35	0.117	0.019
22:5 *n*-6 (DPA)	0.97	0.34	1.00	0.33	0.90	0.22	0.95	0.19	0.047	0.327
22:6 *n*-3 (DHA)	5.64	1.00	5.14	0.91	5.76	0.81	5.61	1.17	0.092	0.016
24:0	0.05	0.02	0.04	0.02	0.04	0.02	0.04	0.03	−0.201	0.351
24:1 *n*-9	0.05	0.03	0.03	0.02	0.04	0.03	0.03	0.03	0.172	0.577

Data are presented as geometric mean and geometric standard deviation (SD). ALA, α-linolenic acid; ARA, arachidonic acid; DGLA, dihomo-γ-linolenic acid; DHA, docosahexaenoic acid; DMA, dimethyl acetal; DPA, docosapentaenoic acid; EPA, eicosapentaenoic acid; GLA, γ-linolenic acid; LA, linoleic acid.
