# Correction: Billeaud et al. “Effects on Fatty Acid Metabolism of a New Powdered Human Milk Fortifier Containing Medium-Chain Triacylglycerols and Docosahexaenoic Acid in Preterm Infants” *Nutrients* 2018, *10*, 690

**DOI:** 10.3390/nu11030680

**Published:** 2019-03-22

**Authors:** Claude Billeaud, Carole Boué-Vaysse, Leslie Couëdelo, Philippe Steenhout, Jonathan Jaeger, Cristina Cruz-Hernandez, Laurent Ameye, Jacques Rigo, Jean-Charles Picaud, Elie Saliba, Nicholas P. Hays, Frédéric Destaillats

**Affiliations:** 1 CIC Pédiatrique 1401 CHU, 33000 Bordeaux, France; claude.billeaud@chu-bordeaux.fr; 2 ITERG, Université de Bordeaux, 33076 Bordeaux, France; c.vaysse@iterg.com (C.B.-V.); l.couedelo@iterg.com (L.C.); 3 Nestlé Health Sciences, 1066 Epalinges, Switzerland; philippe.steenhout@nestle.com; 4 Nestlé Research Centre, 1000 Lausanne, Switzerland; jnthn.jaeger@gmail.com (J.J.); cristina.cruz-hernandez@rdls.nestle.com (C.C.-H.); 5 Nestlé Nutrition R&D, 1800 Vevey, Switzerland; laurent.ameye@nestle.com (L.A.); nicholaspaul.hays@nestle.com (N.P.H.); 6 Department of Neonatology, University of Liège, 4000 Liège, Belgium; j.rigo@ulg.ac.be; 7 Hôpital de la Croix Rousse, Hospices Civils, 69004 Lyon, France; jean-charles.picaud@chu-lyon.fr; 8 Hôpital Clocheville, CHU de Tours, 37004 Tours, France; elie.saliba@univ-tours.fr

The authors wish to make a correction to the published version of their paper [1]. While replying to a Letter to the Editor [2], we re-examined our results and realized that an erroneous version of Table 4 had been accidentally incorporated into the paper. During the manuscript review process, we were asked to reorder the table columns, include the standard deviations, and include all of the *p* values. Due to the substantial nature of these edits, many errors were accidentally introduced into this table. We sincerely apologize for this unfortunate error and regret any subsequent confusion. A corrected version of Table 4 is below.

This change does not impact the overall results or scientific conclusions. The original manuscript will remain online on the article webpage, with a reference to this correction.

## Figures and Tables

**Table 4 (Corrected):** Fatty acid profile (g/100 g of fatty acids) of total plasma phospholipids in preterm infants, receiving human milk fortified with a control (cHMF) or with a new human milk fortifier (nHMF) before and after 21 days of treatment. Estimates of the treatment effect nHMF/cHMF (difference) and the two-sided *p*-values are given for each fatty acid analyzed in the different lipid compartments.

	cHMF (*n* = 21)				nHMF (*n* = 26)					
	Baseline		After 21 Days		Baseline		After 21 Days		Difference	*p* Value
	Mean	SD	Mean	SD	Mean	SD	Mean	SD		
14:0	0.21	0.14	0.22	0.14	0.24	0.13	0.26	0.13	0.184	0.500
15:0	0.13	0.10	0.18	0.26	0.14	0.08	0.12	0.03	−0.071	0.722
16:0	25.00	5.55	22.75	6.21	26.00	3.58	26.46	2.76	0.180	0.020
16:0 DMA	0.63	0.18	0.70	0.17	0.64	0.16	0.74	0.27	−0.134	0.394
16:1 *n*-7	1.24	0.71	1.05	1.19	1.28	0.69	0.93	0.70	0.209	0.188
16:1 *n*-9	0.32	0.14	0.24	0.15	0.30	0.12	0.25	0.13	0.219	0.117
18:0	14.99	2.90	17.25	4.15	14.51	1.65	15.84	1.63	−0.060	0.203
18:0 DMA	0.33	0.12	0.47	0.37	0.35	0.10	0.36	0.14	−0.402	0.040
18:1 DMA	0.29	0.14	0.32	0.11	0.30	0.09	0.30	0.13	−0.288	0.054
18:1 *n*-7	3.07	0.59	2.36	0.62	3.05	0;89	2.59	0.77	0.122	0.015
18:1 *n*-9	14.04	2.16	11.98	3.37	13.90	3.03	12.45	2.97	0.051	0.286
*trans*-18:1	0.30	0.15	0.42	0.16	0.39	0.13	0.40	0.17	−0.112	0.515
18:2 *n*-6 (LA)	12.90	3.20	14.60	2.53	14.03	2.75	14.69	2.55	0.043	0.452
18:3 *n*-3 (ALA)	0.18	0.14	0.11	0.05	0.18	0.10	0.17	0.08	0.219	0.125
18:3 *n*-6 (GLA)	0.22	0.07	0.17	0.06	0.22	0.06	0.16	0.04	0.030	0.689
20:0	0.36	0.29	0.40	0.31	0.38	0.21	0.41	0.18	0.011	0.945
20:1 *n*-9	0.25	0.08	0.31	0.09	0.27	0.10	0.36	0.12	0.156	0.072
20:2 *n*-6	0.47	0.13	0.67	0.46	0.51	0.11	0.53	0.10	−0.112	0.340
20:3 *n*-6 (DGLA)	3.79	0.89	4.04	1.05	3.46	0.72	3.45	0.68	−0.161	0.041
20:3 *n*-9	2.56	1.46	1.90	1.53	2.12	1.19	2.11	1.51	0.089	0.612
20:4 *n*-6 (ARA)	9.81	2.33	10.63	3.54	9.09	2.03	8.26	2.58	−0.211	0.015
20:5 *n*-3 (EPA)	0.84	0.48	0.70	0.25	0.88	0.32	0.97	0.28	0.318	0.006
22:0	0.46	0.39	0.53	0.53	0.53	0.33	0.57	0.27	0.110	0.567
22:1 *n*-9	0.05	0.03	0.05	0.03	0.05	0.02	0.05	0.02	0.048	0.676
22:4 *n*-6	0.46	0.17	0.49	0.29	0.40	0.13	0.35	0.10	−0.257	0.032
22:5 *n*-3 (*n*-3 DPA)	0.46	0.24	0.53	0.31	0.44	0.12	0.40	0.12	−0.217	0.037
22:5 *n*-6 (*n*-6 DPA)	0.49	0.19	0.53	0.25	0.45	0.25	0.50	0.22	−0.005	0.970
22:6 *n*-3 (DHA)	3.60	1.42	3.96	1.94	3.22	0.93	3.54	1.04	−0.050	0.575
24:0	0.39	0.29	0.53	0.80	0.48	0.28	0.43	0.18	−0.056	0.742
24:1 *n*-9	1.50	1.23	1.47	0.83	1.55	0.98	1.77	0.73	0.191	0.368

Data are presented as geometric mean and geometric standard deviation (SD). ALA, α-linolenic acid; ARA, arachidonic acid; DGLA, dihomo-γ-linolenic acid; DHA, docosahexaenoic acid; DMA, dimethyl acetal; DPA, docosapentaenoic acid; EPA, eicosapentaenoic acid; GLA, γ-linolenic acid; LA, linoleic acid.
